# Determination of α1-acid glycoprotein (AGP) concentration by HPLC in patients following local infiltration analgesia for primary total hip arthroplasty and its relation to ropivacaine (total and unbound)

**DOI:** 10.3389/fphar.2023.1145962

**Published:** 2023-06-26

**Authors:** Muhammad Abbas, Manal A. Alossaimi, Abdulmalik S. A. Altamimi, Mai Alajaji, David G. Watson, Sayyed I. Shah, Yasar Shah, Mohammad S. Anwar

**Affiliations:** ^1^ Department of Pharmacy, Abdul Wali Khan University Mardan, Mardan, Pakistan; ^2^ Department of Pharmaceutical Chemistry, College of Pharmacy, Prince Sattam Bin Abdul Aziz University, Al-Kharj, Saudi Arabia; ^3^ College of Pharmacy, King Abdullah International Medical Research Center, King Saud Bin Abdulaziz University for Health Sciences, Riyadh, Saudi Arabia; ^4^ Strathclyde Institute of Pharmacy and Biomedical Sciences, University of Strathclyde, Glasgow, United Kingdom; ^5^ Department of Pharmacy, University of Swabi, Swabi, Pakistan

**Keywords:** HPLC, α1-acid glycoprotein, human plasma, hip joint surgery patients, protein binding

## Abstract

**Introduction:** This study was performed to determine the levels of α1-acid glycoprotein (AGP) in old-age patients undergoing total hip arthroplasty. AGP is considered an acute phase protein produced during the acute phase reaction in the body to various stimuli; their proper monitoring is thus important.

**Methods:** In order to study how AGP concentrations in old age patients change in response to surgical stress (total hip arthroplasty), a high-performance liquid chromatography assay was performed to measure AGP levels. AGP was isolated from the plasma by adding perchloric acid and was analyzed using PLRP-S 4000°A column. The mobile phase consisted of 1 mL TFA/L of water (Solvent A pH 2) and 1 mL TFA/L of acetonitrile (Solvent B). The gradient used was as follows: 0 min 18% B and 82% A, 15 min 60% B and 40% A, and 17 min 60% B and 40% A followed by column re-equilibration for 7 min before the next injection. AGP peak was obtained between 8.8 and 8.9 min. The method was fully optimised according to established guidelines.

**Results:** The data obtained were analyzed on ChromQuest software. AGP concentrations were determined in all samples, including baseline and samples taken at different timed intervals. The peak for AGP was obtained between 8.8 and 8.9 min for both standard AGP and patient plasma. The graphs indicate that AGP concentration in almost all patient samples increased considerably, especially after 4 h and 24 h—for example, initial concentration in patient 1 was 10.36 mg/100 mL but, after 24 h, increased to 23.50 mg/100 mL. There was thus almost a 13 mg/100 mL increase in 24 h, which is confirmed by AGP concentration increasing after various conditions, including surgery. The increased plasma protein binding was comparatively associated with the unchanged free fraction of the drug.

**Conclusion:** This surgically induced increase in AGP concentration resulted in increased plasma protein binding of the drug (ropivacaine), which in turn kept the free portion of ropivacaine stable during the postoperative period.

## 1 Introduction

Drug binding to different proteins in plasma may be significantly important in drug therapy because drug protein binding serves as a depot for the drug and has an influence on the pharmacokinetic and pharmacodynamic parameters and metabolism of the compound (Zeitlinger et al., 2011). The pharmacological action of the drug is only due to the unbound portion of the drug and not to the total drug present in the plasma (Kratochwil et al., 2002; Banker et al., 2003). Thus, the effect of plasma protein binding on the pharmacokinetic and pharmacodynamic parameters needs careful attention. Many cases have been reported where a small change in plasma protein binding resulted in clinically vital changes in the pharmacokinetic parameters of the drug (Schmidt et al., 2010). “Unbound fraction” is the concentration of a drug which is not bound to plasma proteins, and only this concentration of the drug produces the pharmacological effect. This unbound concentration varies during the dosing interval and must be accurately and correctly explained to determine the drug's effect (Roberts et al., 2013). Drug binding to plasma proteins plays a vital role in determining the diffusion rate of the drug between extracellular and intracellular space; therefore, any change in this diffusion affects pharmacokinetic parameters, including the distribution volume and the clearance rate of the drug (Bohnert and Gan, 2013). Generally, in cases of highly protein-bound drugs, a little change in protein binding is responsible for significant changes in the unbound concentration of drug, thereby increasing the clearance and volume of distribution (Bohnert and Gan, 2013; Roberts et al., 2013).

Acute phase proteins (α1-acid glycoprotein—AGP or AAP) are produced during the acute phase reaction in the body to various stimuli and play a role in the defence response shown by the body. The initiation of acute phase reactions in the body prevents any further injury by removing any harmful material and initiating the repair process to restore the organ to normal function. Their levels also inform us about the progression of the disease or inflammatory response by monitoring their concentrations in the blood. The changes in the plasma concentration of these proteins lead to altered drug binding and ultimately affect the pharmacokinetic and pharmacodynamic parameters of the drug (Tesseromatis et al., 2011).

Among the acute phase proteins, AGP is the most important (Rice et al., 1997; Tesseromatis et al., 2011). It is also called orosomucoid (ORM) and is the main protein responsible for binding basic drugs (Wood and Wood, 1981; Kremer et al., 1988; Booker et al., 1996; Fournier et al., 2000; Hochepied et al., 2003; Tesseromatis et al., 2011; Huang and Ung, 2013; Taguchi et al., 2013). AGP was first described by Karl Schmid and Richard J. Winzler and their colleagues in 1950; they considered it an unusual protein because of its low p*I* value, which ranges from 2.8–3.8 and has a comparatively high content of carbohydrate (45% w/w) (Kishino and Miyazaki, 1997; Kremmer et al., 2004). AGP was considered as having the highest carbohydrate content of any protein until the characterisation of galactoglycoprotein in 1980, which has a 76% carbohydrate content (Hellerstein et al., 1985; Fournier et al., 2000; Xuan and Hage, 2005). AGP is also reported as playing a role in drug–drug interactions due to the displacement of drugs and other endogenous molecules from their binding sites, resulting in significant clinical implications (Tesseromatis et al., 2011).

The concentration of AGP in serum increases in various conditions, like post-surgical inflammation due to burn injury, cancer, and various other conditions (Hellerstein et al., 1985; Kishino and Miyazaki, 1997; Larsson et al., 1997; Hochepied et al., 2003; Huang and Ung, 2013; Eckersall and Schmidt, 2014). AGP synthesis mostly occurs in the liver, and then it is secreted by hepatocytes (Tesseromatis et al., 2011; Keser et al., 2021). However, there is also evidence of extra hepatic synthesis, which is production of AGP in places apart from hepatic tissue like human breast epithelial cells, the kidney, adipose tissue, and the spleen, thymus, and heart (Hochepied et al., 2003). There is also variation in the levels of AGP among healthy individuals depending on age, sex, and hormonal variations (Huang and Ung, 2013). The effectiveness of a drug in these cases depends on whether it is acidic in nature or a basic drug, as well on whether the drug binds to the albumin, to which acidic drugs normally bind, or to AGP, to which basic drugs bind. Drug displacement from protein binding sites by another strongly binding drug will also have a significant effect on the concentration of the displaced drug; dose adjustment should be taken into consideration in the use of such drugs (Roberts et al., 2013; Bteich, 2019).

## 2 Methodology

### 2.1 Patients

We measured AGP in 20 patients (12 male and 8 female patients) aged 65 years or over undergoing unilateral total hip arthroplasty following high-volume, high-dose local infiltration analgesia. The study protocol was approved by the Regional Ethics Committee and conducted in accordance with the Helsinki Declaration (NCT01873313) from August 2012 to March 2013 in Golden Jubilee Hospital, Glasgow Scotland. Ten blood samples at different timed intervals were taken from each patient, and AGP was determined. A total of 200 samples were thus analysed for AGP. (20*10 = 200). Local anaesthetic infiltration was performed intra-operatively by the surgeon using 180 mL plain ropivacaine 0.2%. Circumferential infiltration of the deep and peri-capsular tissues with up to 80 mL was followed by infiltration of the gluteal muscles and fascia lata with approximately 70 mL. Finally, 30 mL was used to infiltrate the subcutaneous tissues and skin, completing the total of 180 mL.

### 2.2 Chemicals and blood plasma for AGP analysis

Human AGP (standard), trifluoroacetic acid, and perchloric acid (70%) were purchased from Sigma Aldrich (UK). HPLC-grade water was produced by a Direct-Q 3 Ultrapure Water System from Millipore, UK. HPLC-grade acetonitrile (ACN) was purchased from Fisher Scientific, United Kingdom. Blank plasma was provided by the Blood Transfusion Service of Gartnavel Hospital, Glasgow. Plasma samples were obtained from Dr Mike Gill from patients undergoing hip joint surgery at the Golden Jubilee Hospital in Clydebank. The samples were stored at −20°C until analysis.

### 2.3 HPLC analysis

A high-performance liquid chromatographic (HPLC) system was used in the analysis and consisted of a SpectraSYSTEM P2000 gradient pump, a SpectraSYSTEM AS3000 autosampler (equipped with a Type 7010-150 Rheodyne injection valve (20 µL loop)), and a SpectraSYSTEM UV 1000 detector. The column used in the analysis was a PLRP-S 4000°A column (50 mm × 4.6 mm I.D., 5 µm particle size, Agilent Technologies). The pressure maintained at the column head was about 9,000 psi. The mobile phase consisted of 1 mL TFA/L of water (Solvent A pH 2) and 1 mL TFA/L of acetonitrile (Solvent B). The gradient used was as follows: 0 min 18% B and 82% A, 15 min 60% B and 40% A, and 17 min 60% B and 40% A followed by column re-equilibration for 7 min before the next injection. The flow rate of the mobile phase was 1 mL/min. The detection wavelength was set to 220 nm. The column was kept at room temperature. The data acquired were processed using ChromQuest software.

### 2.4 Standards preparation

Stock solution of AGP was prepared at a concentration of 2 mg/mL in water. Different calibration solutions were then prepared from the stock solution by diluting it in 0.5 M perchloric acid. Different standards at concentrations of 6.25 mg/100 mL (0.0625 mg/mL), 12.5 mg/100 mL (0.125 mg/mL), 25 mg/100 mL (0.25 mg/mL), 50 mg/100 mL (0.5 mg/mL), and 100 mg/100 mL (1 mg/mL) were prepared and directly injected into the chromatographic system.

### 2.5 Sample preparation

Venous blood samples from each patient participating in the study were taken at 5, 10, 15, 20, 25, and 30 min, then 1, 4, and 24 h following the injection of local anaesthetic, at the Golden Jubilee Hospital in Clydebank. The samples were then transferred to the University of Strathclyde, Glasgow, for analysis.

The concentrations of AGP in all the patient plasma were then determined as follows: to 50 µL of patient or control plasma, 100 µL of 0.5 M of perchloric acid was added and was vortexed for 20 S in an Eppendorf tube to mix it thoroughly. The acidified plasma was then centrifuged at 9,000 rpm for 5 min at room temperature. The supernatant was then transferred to the insert in the HPLC vial for analysis. Both the extracted standards and samples were stored at 4°C before being chromatographed.

The AGP concentration was then calculated from its linear correlation versus the values of peak areas obtained from the perchloric acid extract, expressed as mg/100 mL of plasma.

### 2.6 Method validation

The method was validated according to ICH guidelines.

#### 2.6.1 Linearity

The method’s linearity was established using the standards for AGP dissolved in perchloric acid as described in Section 2.4.

#### 2.6.2 Recovery

In order to determine the accuracy of the extraction procedure, the recovery of AGP by perchloric acid extraction was determined. AGP was added to a constant volume of plasma (50 μL) in two different concentrations (25 and 50 mg/100 mL) and then mixed with perchloric acid as before in order to prepare a standard addition curve.

#### 2.6.3 Accuracy and precision

The within-run reproducibility of retention time and peak area was examined by repeated injection (*n* = 3) of an AGP standard (50 mg/100 mL). Three replicates prepared from the 50 μL amounts of the pooled plasma QC sample were run on three different occasions to calculate within- and between-run precision.

#### 2.6.4 Reproducibility

Replicates of the same QC samples (50 mg/100 mL) were run alongside the patient samples to monitor the performance of each assay.

#### 2.6.5 Sensitivity

The sensitivity of the method was determined in standard solutions and blank plasma spiked with the analyte. The limit of detection was defined as the minimum concentration for which a signal-to-noise ratio of 3 was obtained; for LOQ, a signal-to-noise ratio of 10 was considered.

#### 2.6.6 Application of the method to patient plasma

To determine the clinical relevance of the method, the concentration of AGP was identified and determined in the plasma of old-age hip joint surgery patients. The procedure was discussed in Section 2.5.

### 2.7 Ropivacaine determination

A fully validated method was developed on LC-MS to determine the free and bound portions of ropivacaine and published in Abbas et al. (2013).

## 3 Results

The normal mean value for AGP concentration in the plasma is 77 mg/100 mL (Booker et al., 1996). However, there is great variation of this value among individuals. The results we obtained in our analysis gave concentrations which were generally below those specified by previous studies.

### 3.1 Results of standard AGP samples and patient plasma

**FIGURE 1:**
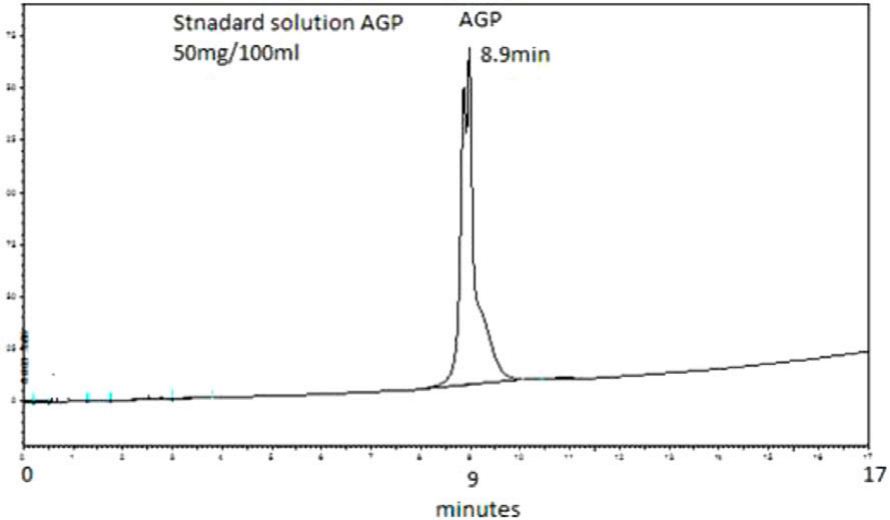
Typical chromatogram of an unextracted aqueous standard containing 50 mg/100 mL of AGP. The elution was performed on a PLRP-S 4000°A column (50 mm × 4.6 mm I.D., 5 µm particle size, Agilent Technologies). The mobile phase consisted of 1 mL TFA/L of water (Solvent A) and 1 mL TFA/L of acetonitrile (Solvent B). The gradient used was 0 min 18% B, 15 min 60% B, and 17 min 60% B followed by column re-equilibration for 7 min before the next injection. The flow rate of the mobile phase was 1 mL/min. The detection wavelength was set to 220 nm. Retention time observed was 8.9 min.

**FIGURE 2:**
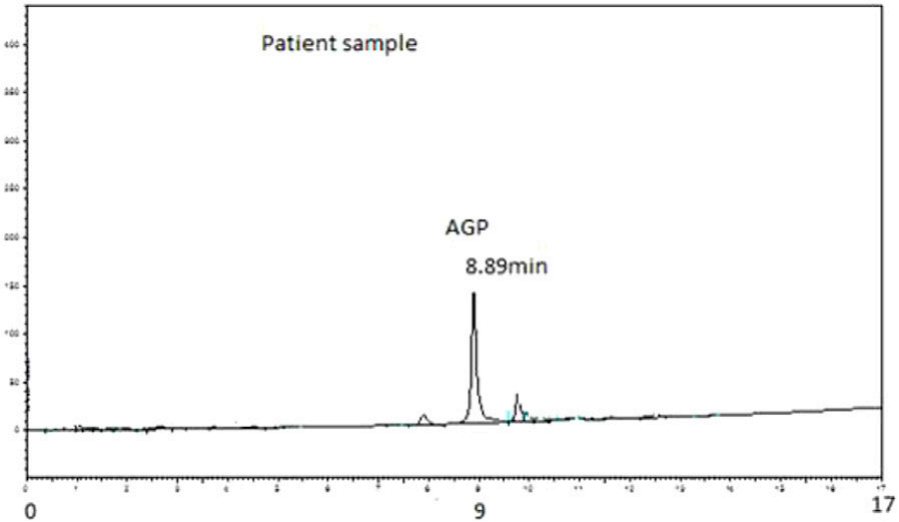
Typical chromatogram of AGP extracted from 50 µL of patient plasma. Analysis was performed on PLRP-S 4000°A column (50 mm × 4.6 mm I.D., 5 µm particle size, Agilent Technologies). The mobile phase consisted of 1 mL TFA/L of water (Solvent A) and 1 mL TFA/L of acetonitrile (Solvent B). The gradient used was 0 min 18% B, 15 min 60% B, and 17 min 60% B followed by column re-equilibration for 7 min before the next injection. The flow rate of the mobile phase was 1 mL/min. The detection wavelength was set to 220 nm. Retention time observed was 8.89 min.

The AGP concentrations were analysed in the plasma of 20 hip joint surgery patients. These samples were taken at 5 min, 10 min, 15 min, 20 min, 25 min, 30 min, 1 h, 4 h, and 24 h. Figure 1 shows a typical chromatogram of the AGP standard injected directly into the chromatographic system, and Figure 2 shows a chromatogram of the AGP in patient plasma following removal of the rest of the protein with 0.5 M perchloric acid. The peak for AGP was obtained between 8.8 and 8.9 min. This method works very well for determining AGP, and there is minimal interference from other serum proteins like HSA, which is precipitated out almost completely by the perchloric acid; AGP remains in solution because of its high water solubility, which is due to its 40% carbohydrate content (Stumpe et al., 2006).

### 3.2 Method validation

#### 3.2.1 Linearity

The calibration curve obtained by plotting the areas of the AGP peaks versus AGP concentration on different days was linear (calibration curve added in Supplementary Material). The data were obtained for the calibration curve on three different days, and the average of these data was used for the calibration curve. The curve covered the concentration range from 6 mg/100 mL to 100 mg/100 mL, which also covered the concentration of AGP in the patient plasma.

#### 3.2.2 Recovery

In the recovery test, the recovery after liquid–liquid extraction with perchloric acid of 25 mg/100 mL spiking of AGP into plasma was 91.7% ± 8.9% (*n* = 6), and, for 50 mg/100 mL spiking, the recovery was 90.1% ± 3.7% (*n* = 6).

#### 3.2.3 Accuracy and precision

The within-run precision of the extraction/HPLC procedure ranged from ±1.2% to ±3.8%, and the between-run precision was ±6.1%.

#### 3.2.4 Reproducibility

Having established linearity, a single-point standard of 50 mg/100 mL was run every day with the patient samples, and results were always well within limits.

#### 3.2.5 Sensitivity

The method was sensitive enough to determine the lowest level of AGP 5 mg/100 mL (LOD), while LOQ was found to be 10 mg/100 mL.

#### 3.2.6 Application of the method to patient plasma

The results for the AGP concentrations in patients receiving a continuous infusion of a local anaesthetic ropivacaine following total hip arthroplasty are presented in Figure 4.

## 4 Discussion

The margin of therapeutic safety for many drugs like anaesthetics should be considered in older age because the body's ageing process may result in toxicity of the drugs. Very little data have been published regarding the binding of local anaesthetics to plasma proteins. This study was performed to determine the level of AGP in the plasma of old-aged hip joint surgery patients.

The level of AGP in the plasma increased during the study period, which indicated that the level of AGP increases during or following surgical procedures. Similar results were shown in a previous study (Bohnert and Gan, 2013) which determined the concentration of AGP in neonates and also by two other studies Hellerstein et al. (1985) and Tesseromatis et al. (2011).

**FIGURE 3:**
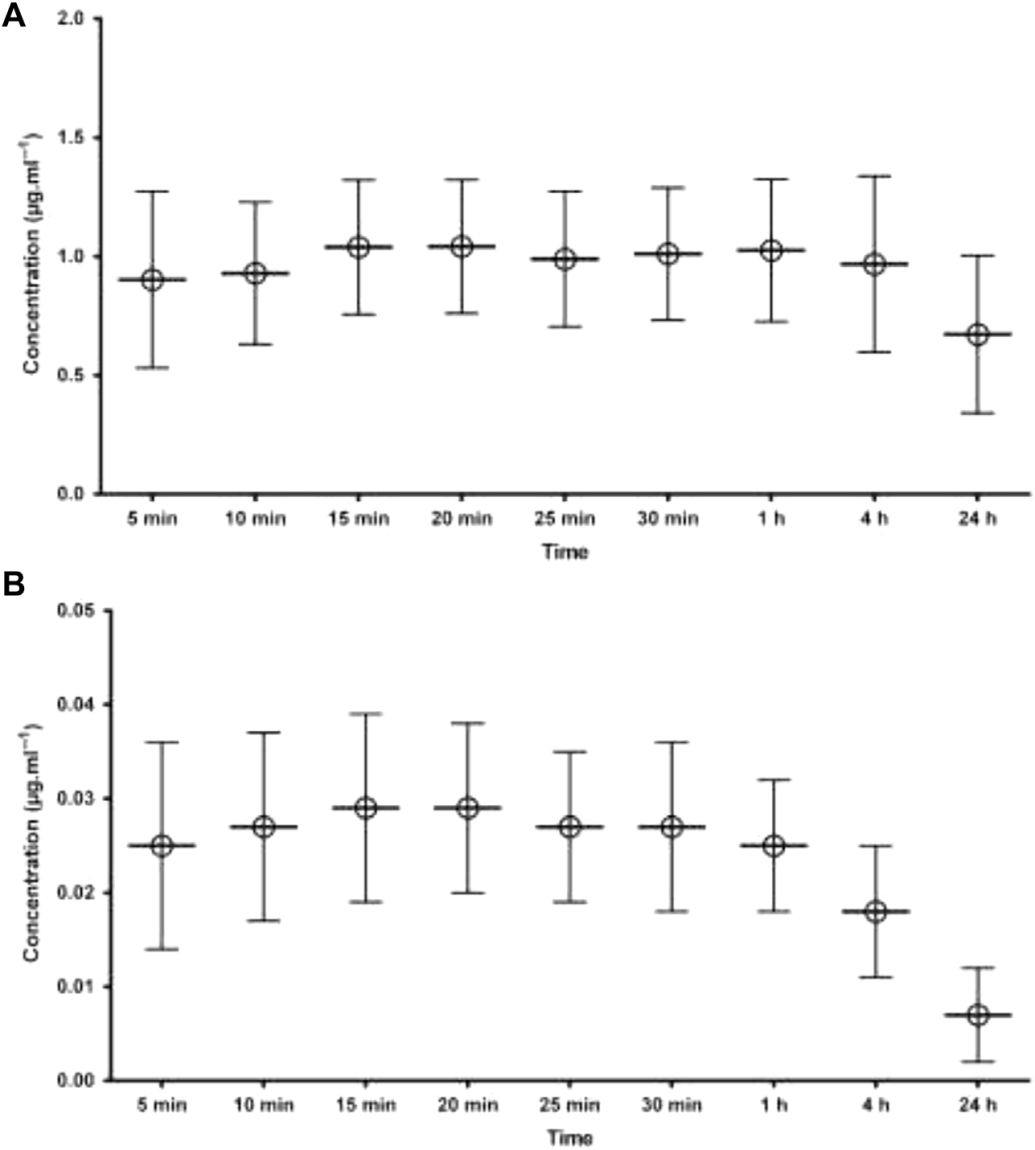
Mean plasma concentration of ropivacaine following local infiltration analgesia **(A)** total concentration i.e., bound to AGP **(B)** free concentration of ropivacaine. The error bars indicates SD.

**FIGURE 4:**
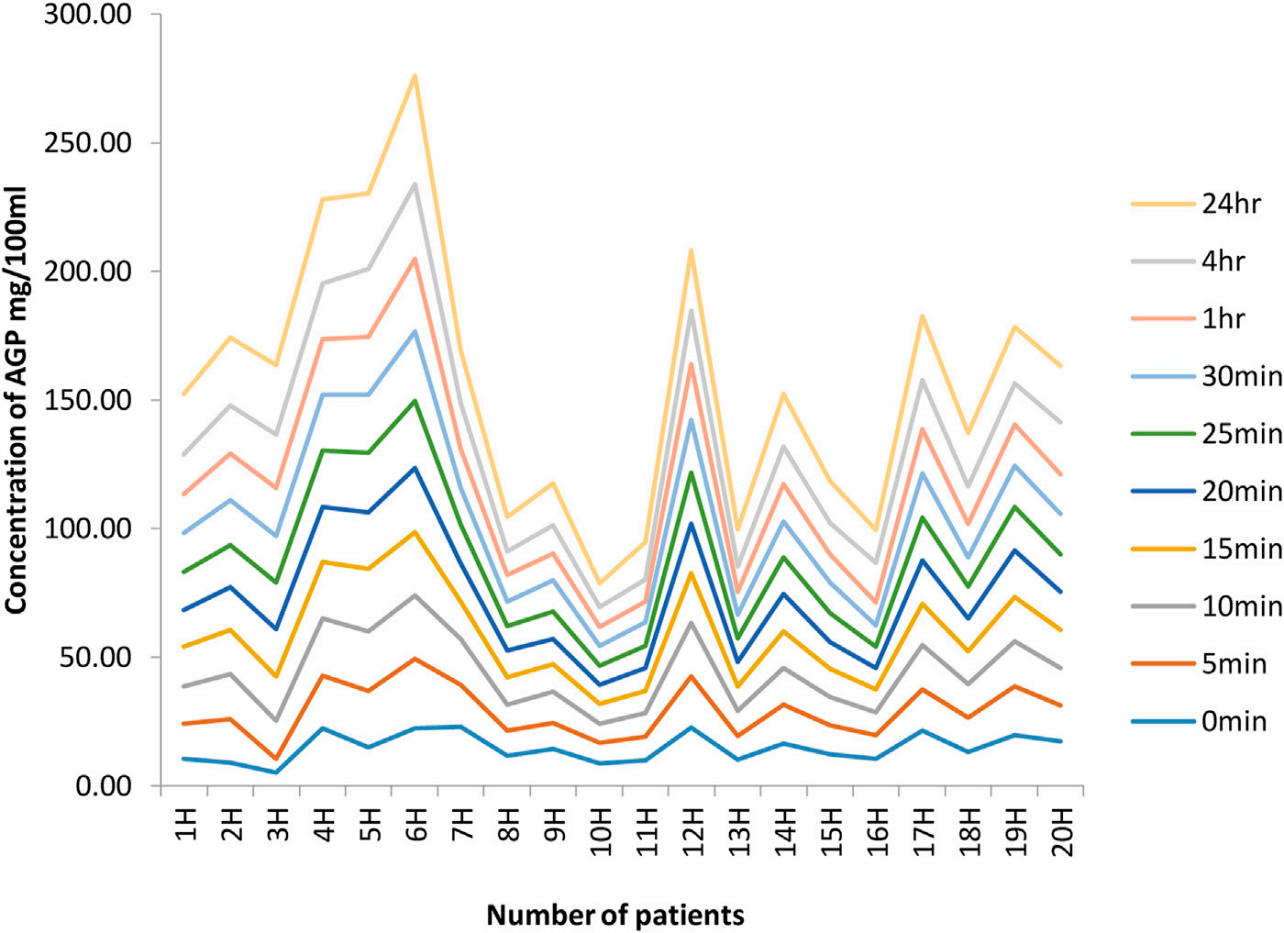
Concentrations of AGP at different time intervals in 20 hip joint patients. The graphs indicate a significant increase in AGP concentration after surgery, especially in samples taken after 4 h and 24 h. In patient 1, initial concentration is 10.36 mg/100 mL, but, after 24 h, it increases to 23.50 mg/100 mL. There is thus almost a 13 mg/100 mL increase in 24 h. In patient 2H, there is also a significant increase in the initial concentration, and, after 24 h, the table and from graph show it as 8.8 mg/100 mL and, after 24 h, 26.44 mg/100 mL. In patient 3H, there is also a considerable increase in initial concentration of 5.19 mg/100 mL; after 24 h, it is 27.05 mg/100 mL. Likewise, there is an increase in AGP concentration in all 20 patients.

As can be seen from graphs of the results (Figure 4), nearly all patients had AGP levels which were higher after 4 h and 24 h than early timed samples. This increase in AGP level is directly related to plasma protein binding, which increases in the samples taken after 24 h. The increased plasma protein binding was comparatively associated with the unchanged free fraction of the drug. As the effect of the drug is mainly due to its free or unbound portion, any altered concentration of AGP will ultimately affect the level of the unbound portion of the drug and its subsequent effect. This surgically related increase in AGP concentration resulted in increased plasma protein binding of the drug (ropivacaine), which in turn kept the free portion of ropivacaine stable and within limits during the postoperative period, as shown in Figure 3. A detailed method regarding ropivacaine determination was published in Abbas et al. (2013).

The data and graphs in Figure 4 clearly show a significant increase in AGP concentration after surgery, especially in samples taken after 4 h and 24 h. In patient 1, the initial concentration is 10.36 mg/100 mL, but, after 24 h, it increases to 23.50 mg/100 mL. There is thus almost a 13 mg/100 mL increase in 24 h. In patient 2H, there is also a significant increase in initial concentration and after 24 h, as seen in the table and from the graph, of 8.8 mg/100 mL and, after 24 h, 26.44 mg/100 mL. In patient 3H, there is also a considerable increase in the initial concentration of 5.19 mg/100 mL and, after 24 h, 27.05 mg/100 mL. There is likewise an increase in the concentration of AGP in all 20 patients. This confirms that there is an increase in AGP concentration after surgical conditions as well as in stress conditions as shown in different studies.

It is also clear from the data that there is considerable inter-individual variation in the level of AGP. It was found by Booker et al. (1996) that the mean concentration is 77 mg/100 mL; however, based on our results, it is difficult to determine any exact concentration because of this inter-individual variability in AGP concentration. Therefore, this is a solid base for any dose adjustment in the case of surgical condition because of increased levels of AGP.

## 5 Conclusion

The results confirmed that AGP concentration in almost all patients increased considerably in all samples, especially after 4 h and 24 h, which confirms that AGP concentration increases after surgical conditions. Therefore, dose adjustment should be considered in the use of drugs which bind to AGP.

## Data Availability

The original contributions presented in the study are included in the article/Supplementary Material; further inquiries can be directed to the corresponding authors.
