# Supplementary figures and images for: Determination of α1-acid glycoprotein (AGP) concentration by HPLC in patients following local infiltration analgesia for primary total hip arthroplasty and its relation to ropivacaine (total and unbound)
Source: Front Pharmacol. 2023 Jun 26;14:1145962. doi: 10.3389/fphar.2023.1145962 (PMC10345198; doi:10.3389/fphar.2023.1145962)

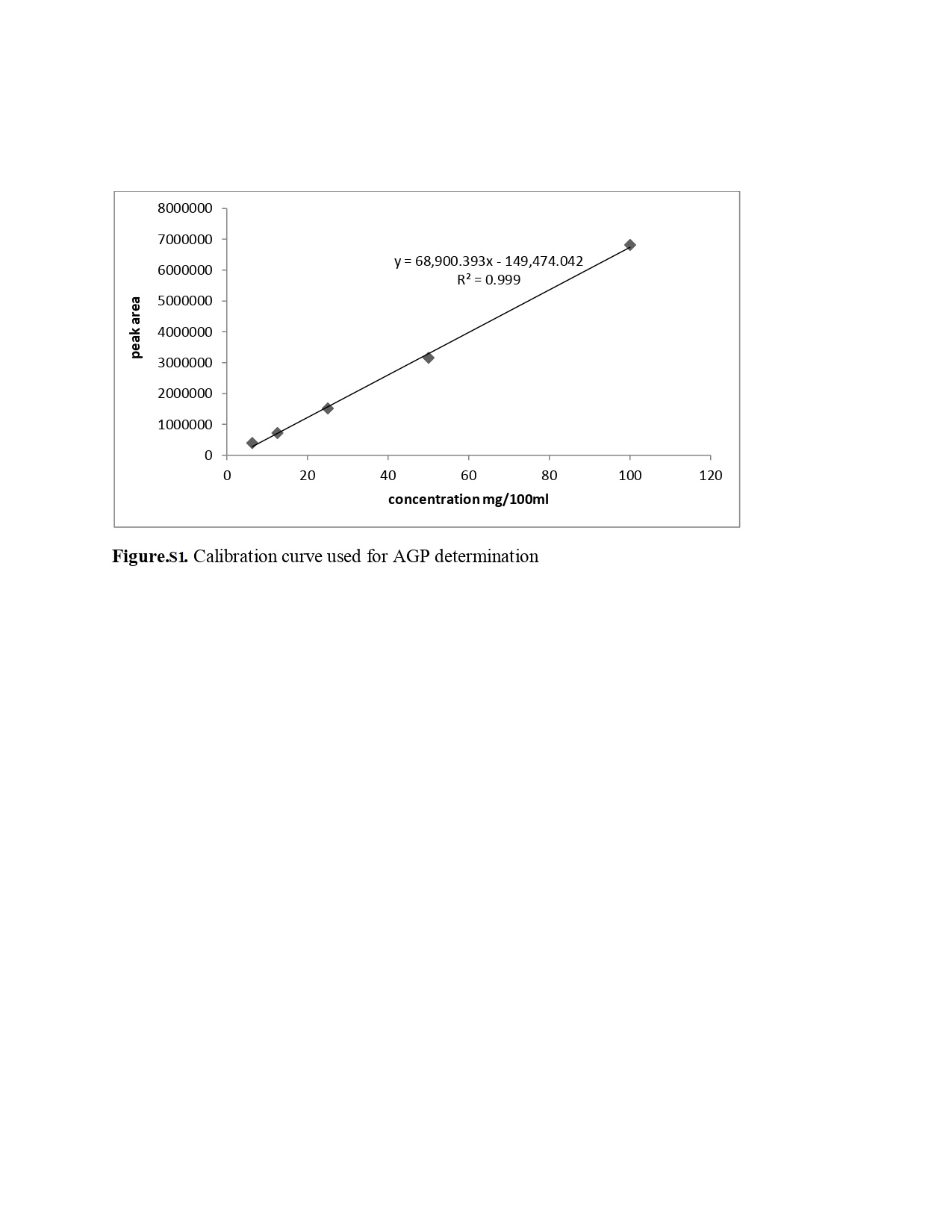

Supplement: Supplementary file 1 [file Image1.jpg]
